# Onset of the Non-Medical Use of Prescription and Over-the-Counter Medications during Early Adolescence: Comparison with Alcohol, Tobacco, and Marijuana

**DOI:** 10.3390/children10081298

**Published:** 2023-07-28

**Authors:** Sarosh Khan, Kenneth W. Griffin, Gilbert J. Botvin

**Affiliations:** 1 Department of Psychiatry, Boston University Medical Center, Boston, MA 02118, USA; sarosh.khan@bmc.org; 2 Department of Global and Community Health, George Mason University, Fairfax, VA 22030, USA; 3 Department of Population Health Sciences, Weill Cornell Medical College, New York, NY 10021, USA; gjbotvin@med.cornell.edu

**Keywords:** non-medical medication use, substance use, adolescents, marijuana, alcohol, cigarettes

## Abstract

This study examined the prevalence and psychosocial predictors of the non-medical use of prescription and over-the-counter (OTC) medications and compared these to cigarette, marijuana, and alcohol use in a cohort of early adolescents (*N* = 1887) aged 11 to 13, a critical risk period for the initiation of substance use. Participants were students attending 22 middle schools in the northeastern United States. Participants completed surveys in the classroom, the first in the sixth grade and a second in the seventh grade, and the rate of overall substance use more than doubled from 5.5% to 11.9% over this period. Predictors of the onset of non-medical prescription and over-the-counter drug misuse overlapped substantially with those for marijuana and other substances. The perception of friends’ substance use and the belief that substance use can help you deal with problems predicted the onset of marijuana use, OTC medication misuse, and prescription drug misuse. Decision-making skills were protective for the onset of all substance use outcomes. The findings of this study have important implications for prevention and suggest that a single comprehensive approach may be sufficient for preventing multiple forms of substance use onset during early adolescence.

## 1. Introduction

Several prescription medications are used for non-medical and recreational purposes in the United States, including opioids and other pain relievers, sedatives and tranquilizers, and stimulants. Over-the-counter (OTC) medications sold without a prescription are also often misused, including codeine-based medicines, cough products, sedative antihistamines, decongestants, and laxatives [1]. The trends and prevalence of non-medical prescription and OTC medications among early adolescents have been understudied. Therefore, it is important to investigate how they can vary across social and demographic subgroups, as well as identify key psychosocial risks and protective factors for these behaviors.

The Monitoring the Future (MTF) study reports several key findings about medication misuse among adolescents attending secondary schools in the USA [2]. In 2022, the annual prevalence of OxyContin and Vicodin without a doctor’s order was 2% and 1.3% or less among 8th–12th graders (ages 13–18), and 3% of these students reported using amphetamines in the last year without a doctor’s order. Among 12th graders (high school seniors), 1.1% reported using Ritalin without a doctor’s order in 2022, while less than 1% of 8th and 10th graders reported past year Ritalin use without a prescription. The annual prevalence of non-medical use of Adderall among 10th and 12th graders was 2.9% and 3.4% in 2022 [2].

The prevalence rates and factors affecting the initiation of substance use in younger adolescents are significant because earlier onset of substance use is linked to later, more serious levels of substance use involvement [3,4]. Previous studies have found that adolescents who initiate regular use of alcohol during adolescence are significantly more at risk for developing an alcohol use disorder [5,6]. Initiating cigarette smoking early in life can rapidly lead to nicotine dependence among some youth [7], increasing the risk of developing a lifelong smoking habit. Opioid use during secondary school is associated with an increased risk of long-term opioid use and possible misuse in adults [8]. Generally, early substance use onset is associated with many long-term adverse effects, including negative social, health, and behavioral outcomes; physical and mental health problems; violent and aggressive behaviors; adjustment problems in the workplace; and family problems [9].

Etiologic risk and protective factors occur at multiple levels of influence, including at the individual, family, community, and societal levels, and some factors are more amenable to intervention than others. Some of the most effective substance abuse prevention approaches that focus on individual-level factors incorporate a combination of social resistance skills training, normative education, and competence enhancement skills training. Social resistance interventions aim to increase a student’s awareness of social influences that support substance use, including ways to identify social situations where they may be pressured to smoke or drink. Social resistance interventions also build students’ skills so they can effectively resist peer pressures to smoke, drink, or use substances. Competence enhancement interventions teach cognitive skills to increase self-control, self-esteem, coping strategies, and social skills. Normative education interventions aim to correct inaccurate and often inflated beliefs about the prevalence and acceptability of substance use among friends, peers, and/or adults. The interplay between developmental vulnerabilities and social influences to engage in substance use may promote adolescents’ drug use behaviors; therefore, intervention strategies often target risk and protective factors that are salient in accordance with developmental factors and social influences [10,11].

Some correlates of non-medical use of prescription medications are school performance and involvement, history of a major depressive episode, individual-level characteristics such as risk-taking, sensation seeking, low impulse control, parental and peer attitudes, other illicit drug use, and delinquency [12,13,14,15,16]. Adolescents’ risk for lifetime OTC abuse is lower for those involved in pro-social behaviors, educated about drug use through teachers, schools, or parents, and those with parents and peers who disapprove of youth drug use [17]. Many of these psychosocial factors are common to the initiation of overall substance use among adolescents [18,19,20,21,22,23].

The goal of the present study was to examine the prevalence and onset of misusing prescription and OTC medications in a group of middle school students in the sixth and seventh grades (ages 11–13) and compare these to rates of cigarette smoking, marijuana use, and drinking alcohol, which have been studied more widely. In addition, we examined the extent to which variables targeted by contemporary prevention programs are associated with the early onset of prescription and OTC medication misuse during this critical developmental period in comparison to how they are associated with cigarette smoking, marijuana use, and drinking alcohol. These variables included individual skills, perceived social benefit of substance use, and normative expectations regarding friends’ and peers’ substance use behaviors. Few existing studies have examined predictors of the onset of non-medical prescription drugs and over-the-counter drug misuse during early adolescence, a critical risk period for the initiation of substance use. If the predictors of non-medical prescription drug and over-the-counter drug misuse overlap with those for alcohol, tobacco, and marijuana use, then this would have important implications for prevention and suggest that a single comprehensive approach may be effective for preventing multiple forms of substance use onset during early adolescence.

## 2. Materials and Methods

A total of 1887 students from twenty-two middle schools in the northeast U.S. completed surveys in the classroom at two time points over a two-year period. The sample was 75% white, 10% Hispanic or Latino, 4% black, 3% Asian, and 8% biracial or other. The sample included 54% girls and 46% boys. About 74% of these students lived in two-parent households.

### 2.1. Procedure

Students completed a self-report questionnaire in the 6th grade (ages 11–12) and were followed up and completed a second survey in the 7th grade (ages 12–13). The questionnaire included measures for the frequency of substance use and several psychosocial and demographic variables. The staff ensured students of confidentiality and that students’ responses would not be shared with parents, teachers, or school personnel. Students were also assured that each survey was coded with a unique identifier available only to the researchers and were instructed not to put their names anywhere on the survey. A team of data collectors who were members of the same racial/ethnic groups as the participating students administered the questionnaires during a regular classroom period.

### 2.2. Measures

The survey measures were developed and used in several large substance abuse and violence prevention trials with diverse samples of adolescents funded by the U.S. National Institutes of Health [24,25,26], and the measures have been shown to have excellent psychometric properties [27,28].

*Substance use* was assessed by asking students how much they “smoke cigarettes”; “drink beer, wine, wine coolers or hard liquor”; “smoke marijuana or hashish”; “take someone else’s prescription medication to get high”; and “use over-the-counter medications to get high”. Students were asked to record their responses on a nine-point scale from never to more than once a day. The onset of use for each substance was determined when a student reported no use in the 6th grade and any use in the 7th grade for a particular substance.

In addition to demographic factors, including gender, belonging to a minority race, and belonging to a two-parent household, the predictor variables examined in the present study included (1) perceived social benefit of substance use (e.g., substance use makes you look cool); (2) perceived normative expectations, including students’ perception of how much their friends and peers use these substances; and (3) individual skills including decision-making and drug refusal skills.

*Perceived social benefits* of substance use (α = .86) were assessed with several items with response options ranging from (1) strongly disagree to (5) strongly agree regarding statements about whether cigarette smoking, drinking alcohol, and smoking marijuana “make you look more grown up”, “makes you look cool”, “is a good way of dealing with problems”, “lets you have more fun”, and “have more friends”. Student responses were recoded so that (4) agree and (5) strongly agree were collapsed together to make the variable dichotomous in order to measure how effectively the perception of the social benefit of substance use played a role as a risk factor for the onset of use.

*Perceived normative expectations* (α = .87) regarding the prevalence of peers’ and friends’ smoking cigarettes, drinking alcohol, and smoking marijuana were assessed, with response options ranging from (1) none to (5) all or almost all. Their ratings of (4) more than half and (5) all or almost all were categorized together to make the variable dichotomous to measure how high normative expectations of substance use among friends and peers served as a risk factor for onset.

*Decision-making skills* were assessed using five items (α = .89) for which students rated their responses on a five-point scale from never to always on statements such as “When I have a problem or need to make an important decision, I get the information needed to make the best choice”. Student responses were recorded, and their ratings of almost always and always were categorized together to make the variable dichotomous in order to measure how effectively decision-making skills were protective of substance use onset.

*Drug-refusal skills* (α = .88) were assessed using students’ likelihood to “say no” when offered to use any of the substances on a 5-point Likert scale ranging from (1) definitely would say “no” to (5) definitely would not say “no”. Student responses were recoded so that (4) probably would not say “no” and (5) definitely would not say “no” were categorized together to make the variable dichotomous in order to measure how effectively drug-refusal skills were protective of substance use onset.

## 3. Results

We used *t*-tests and chi-square analyses to examine prevalence rates and gender differences in substance use, followed by a series of logistic regression analyses examining the extent to which the onset of each substance was predicted by skills variables, perceived benefits, and normative expectations.

### 3.1. Prevalence Rates of Substance Use

As shown in Table 1, alcohol was the most widely used substance reported among sixth graders (3.2%) and seventh graders (9.7%). In the sixth grade, males (1.0%) were significantly more likely than females (0.2%) to report cigarette use (t(1791) = 2.14, *p* < 0.05), and males (7.4%) were significantly more likely than females (3.9%) to report any substance use (t(1774) = 3.26, *p* < 0.001). In the seventh grade, males (11.5%) were significantly more likely than females (8.1%) to report alcohol use (t(1791) = 2.43, *p* < 0.01). Alcohol use was initiated by 7.6% of students between grades six and seven. Other than alcohol, fewer than 1% of sixth graders reported the use of the remaining substances.

In the seventh grade, alcohol remained the most prevalent form of substance use. Almost three times as many students reported alcohol use in the seventh grade (9.7%) relative to the sixth grade (3.2%). As shown in Table 2, between grades six and seven, 2.2% of students reported the onset of smoking cigarettes, 2.1% reported the onset of using marijuana, 0.9% reported the onset of using over-the-counter medication, and 0.8% reported the onset of using prescription medication to become high. Overall, 12.3% of males and 10.2% of females initiated the use of any substance from grades six to seven.

### 3.2. Individual Skills, Perceived Social Benefits, and Normative Expectations

For individual skills, decision-making skills and drug refusal skills were analyzed. Almost 80% of students reported that they “almost always” or “always” utilize decision-making skills, such as obtaining the information they need to understand the problem and thinking about their choices before making a decision. In terms of drug refusal skills, a small proportion of the sample responded that they would not be sure or would definitely not say “no” if offered to smoke a cigarette (4.5%), drink beer (8.9%), smoke marijuana (4.3%), take someone else’s prescription medication, or use OTC medications to become high (0.6%). Thus, these scores indicate that the skill levels for these variables were generally high in the sample.

Overall, few students perceived there to be many social benefits of substance use. Boys were more likely to report that there were social benefits associated with using these substances. In grade six, the greatest perceived social benefit of substance use amongst boys (7.8%) and girls (5.7%) was that these substances make one look more grown up. In grade seven, the greatest perceived social benefit of substance use among boys (3.8%) and girls (3.1%) was that these substances let them have more fun.

Less than 10% of the sample expected that their friends smoke cigarettes (7.4%), drink alcohol (8.7%), or use marijuana (3.0%). A greater number of students expected that their peers (other than “friends”) smoke cigarettes (37.7%), drink alcohol (37.4%), or use marijuana (23.1%). An even greater number of students expected that “more than half” or “all or almost all” adults smoke cigarettes (38.3%), drink alcohol (52.6%), or use marijuana (11.3%). Taken together, the majority of students reported good decision-making and drug refusal skills and relatively low levels of perceived benefits of substance use. However, when their estimates are compared to national data, students reported elevated normative expectations of friends’ substance use, particularly for cigarettes, alcohol, and marijuana use.

### 3.3. Logistic Regression Analyses

We conducted a series of logistic regressions to examine how the set of psychosocial predictors assessed in the study predicted the onset of each substance and overall substance use. As shown in Table 3, the initiation of multiple forms of substance use was associated with poor decision-making, high perceived substance use by friends, and beliefs that those who used substances had more friends.

Further analyses examined the predictors of onset for each individual substance. Stronger decision-making skills were protective for the onset of drinking alcohol (OR = 0.32 (0.21, 0.49), *p* < 0.01), the most prevalent substance used. The key risk factors associated with initiating drinking alcohol were misperceptions that drinking alcohol was normative (friends’ use, OR = 2.49 (1.49, 4.18), *p* < 0.01; peer use, OR = 1.22 (1.04, 1.42), *p* < 0.05) and that drinking enables students to have more friends (OR = 3.04 (1.01, 9.22), *p* < 0.05). Student attitudes about the association between cigarettes and “looking cool” served as a risk factor for the onset of smoking cigarettes (OR = 20.60 (1.46, 292.70), *p* < 0.05). Decision-making (OR = 0.22 (0.11, 0.46), *p* < 0.001) and students’ drug refusal skills (OR = 0.26 (0.10, 0.74), *p* < 0.05) were protective for the onset of cigarette use. Decision-making skills (OR = 0.25 (0.12, 0.50), *p* < 0.001) and drug refusal skills (OR = 0.19 (0.05, 0.67), *p* < 0.01) were also protective for the onset of marijuana use. Student perception that marijuana use was effective in “dealing with problems” (OR = 14.32 (1.81, 113.28), *p* < 0.01) and their perception that a majority of friends use substances (OR = 2.89 (1.30, 6.42), *p* < 0.01) were risk factors for initiating marijuana use.

The significant predictors of prescription or OTC medication misuse onset were decision-making skills which were protective (OR = 0.18 (0.07, 0.48), *p* < 0.01), and two factors that increased risk were perceived norms regarding friends’ substance use (OR = 5.13 (1.80, 14.60), *p* < 0.01) and the belief that substance use can help you deal with problems (OR = 12.90 (1.02, 164.80), *p* < 0.05). Generally, predictors of the onset of non-medical prescription and over-the-counter drug misuse overlapped substantially with those for marijuana and other substances. One exception was that while drug refusal skills were protective for cigarette and marijuana use, they were not associated with prescription or OTC medication misuse. This finding is likely due to a lack of variability and ceiling effects, where virtually all students (over 99%) reported that they would refuse offers of non-medical prescription and OTC medications.

## 4. Discussion

In the present study, we examined the prevalence and onset of prescription and OTC medication misuse among boys and girls during the “tween” years of early adolescence, a critical developmental period for the initiation of risk behaviors. We also examined the prevalence and onset of cigarette, alcohol, and marijuana use to make a comparison between rates of initiation across substances. We sought to characterize participants with regard to a variety of potentially important individual-level psychosocial variables that may be linked to the early onset of substance use, including decision-making and drug refusal skills, perceived social benefits, and normative expectations regarding substance use. These etiologic factors are targeted by some contemporary evidence-based prevention approaches.

In this sample, the prevalence rate of using any substance more than doubled, from 5.5% to 11.9% for students going from grade six to seven; furthermore, 12.3% of males and 10.2% of females reported initiating use of any substance from grades six to seven. These findings suggest that the period between grades six and seven, roughly ages 11 to 13, is an important time to intervene with prevention programming. The risk periods for drug use are typically associated with major transitions such as puberty, advancing from elementary to middle school and later to high school, as adolescents experience more social, academic, and psychological challenges [29,30].

Findings indicated that alcohol use was the most commonly used substance in both sixth and seventh grades. The initiation of drinking alcohol and cigarette smoking was the most prevalent, with 7.6% of students reporting initiating alcohol use and 2.2% reporting initiating cigarette smoking between grades six and seven. The initiation of cigarette smoking and marijuana use was about twice as much as the initiation of prescription (0.8%) and OTC medication (0.9%) misuse. The finding that alcohol onset had the highest prevalence is consistent with national data provided by the MTF, which found that alcohol prevalence continued to be the highest among secondary school students. Alcohol and cigarettes may be among the most widely used substances in early adolescents because they are more widely available than illegal substances. The MTF study showed that in 2022, eighth graders perceived alcohol and cigarettes as being easily available: 42% of eighth graders reported “fairly easy or very easy” access to alcohol; 34% reported “fairly easy or very easy” access to cigarettes; compared to only 8% who reported “fairly easy or very easy” access to sedatives or barbiturates [2].

In terms of etiologic factors, findings indicated that some skills were more widely protective than others. Strong decision-making skills were found to be significantly protective against the initiation of prescription and OTC medications, as well as alcohol, cigarettes, and marijuana. Decision-making, as measured in this study, incorporated aspects of obtaining information, problem-solving, and critical thinking. These findings further attest to the importance of a cognitive-behavioral approach to prevention strategies [11]. Drug refusal skills were found to be protective for the onset of cigarette smoking and marijuana use, but not alcohol use or the misuse of prescription or OTC medications.

Specific perceived social benefits were linked to the onset of particular substances. Students who believed using substances could help them “deal with problems” were more likely to begin using marijuana as well as non-medical prescription and OTC use in grade seven. The perceived potential social benefits of prescription and OTC medications as means of problem-solving, as well as the association with decision-making, should be considered in light of recent findings from samples of older adolescents and young adults. In a sample of 7th- through 12th-grade students, illicit use of medications was generally used for self-medication for sleeping or anxiety problems or self-treatment of pain [31]. In a sample of undergraduate students, the most commonly reported motives for illicit medication use were to help with concentration, studying, becoming high, and experimentation [32]. In a sample of young adults aged 18–25 years, OTC medication misuse was associated with hopelessness and symptoms of depression, anxiety, and somatic distress [33]. These findings among older adolescents and young adults offer evidence that prescription and OTC medications are misused to self-medicate for problems with pain, anxiety, other mental health symptoms, and sleep. The present findings add to the literature by showing that perceived benefits of substance use play a role in the onset of non-medical prescription and OTC medication use during the early years of adolescence.

In addition, students who believed that alcohol use “lets you have more friends” were more likely to begin drinking; those who believed that smoking cigarettes makes you “look cool” were more likely to begin smoking. The most commonly reported social benefit derived from substance use was the perception that using these substances makes one look more grown up. Decision-making skills and the reported social benefit of having more friends were most consistently predictive across substance use outcomes. Furthermore, the initiation of multiple substances was predicted by decision-making skills, student perceptions of how many of their friends and peers used these substances, and beliefs that those who engaged in substance use “had more friends”.

The present study has several strengths as well as some limitations. The strengths include the longitudinal design among a sample of early adolescents and the use of standardized self-report surveys and measures with well-established psychometric properties. Limitations include the possibility of underreporting of sensitive behaviors, a predominantly white sample, and no information on students’ socio-economic backgrounds. Future research should examine these issues among racial/ethnic samples of youth from diverse socio-economic backgrounds.

## 5. Conclusions

The present study indicates that several individual-level variables targeted by skills-building prevention programs for adolescent substance use are indeed associated with the onset of multiple substances during the years of early adolescence, in the absence of intervention, including prescription and OTC medication misuse. Findings may potentially inform the refinement of existing prevention efforts that aim to prevent the onset of substance use prior to the escalation of use during the years of adolescence and the transition to young adulthood. These findings suggest that broad-based prevention programs may be effective for preventing prescription and OTC medication misuse, and thus separate interventions are not needed to prevent these behaviors. Given the similarity in predictors across multiple substances observed in the present study, an implication of these findings is that a single comprehensive prevention approach that targets common risk and protective factors related to enhancing life skills, social resistance skills, and correcting normative expectations may be effective for preventing multiple forms of substance use onset during early adolescence.

## Figures and Tables

**Table 1:** Prevalence rates of different types of substance use among 6th and 7th graders (ages 11–13; *N* = 1887).

	Grade 6 (Age 11–12)			Grade 7 (Age 12–13)		
	Boys	Girls	Total	Boys	Girls	Total
Alcohol use	3.9%	2.6%	3.2%	11.5% *	8.1% *	9.7%
Cigarette use	1.0% *	0.2% *	0.6%	2.2%	2.6%	2.4%
Marijuana use	0%	0.3%	0.2%	2.0%	2.2%	2.1%
Over-the-counter medication misuse	0.5%	0.2%	0.3%	1.2%	0.6%	0.9%
Prescription medication misuse	0.1%	0.1%	0.1%	1.0%	0.7%	0.8%
Any substance	7.4% *	3.9% *	5.5%	13.5%	10.6%	11.9%

Note: * *p* < 0.05.

**Table 2:** Onset of different types of substance use among 6th and 7th graders (ages 11–13; *N* = 1887).

	Boys	Girls	Total
Onset of alcohol use	10.3%	7.7%	7.6%
Onset of cigarette use	2.4%	2.6%	2.2%
Onset of marijuana use	2.6%	2.5%	2.1%
Onset of over-the-counter medication misuse	1.2%	0.6%	0.9%
Onset of prescription medication misuse	0.7%	1.0%	0.8%
Onset of any substance use	12.3%	10.2%	11.2%

**Table 3:** Odds ratios and 95% confidence intervals from logistic regression analyses predicting the onset of cigarette smoking, alcohol use, marijuana use, prescription, and over-the-counter (OTC) medication misuse, and overall substance use among 6th and 7th graders (ages 11–13; *N* = 1887).

	Onset of Alcohol Use		Onset of Cigarette Use		Onset of Marijuana Use		Onset of Prescription or OTC Misuse	
	OR	95% CI	OR	95% CI	OR	95% CI	OR	95% CI
*Individual Skills*								
Decision Making	0.32 ***	(0.21, 0.49)	0.22 ***	(0.11, 0.46)	0.25 ***	(0.12, 0.50)	0.18 **	(0.07, 0.48)
Drug Refusal	0.63	(0.35, 1.14)	0.26 *	(0.10, 0.74)	0.19 **	(0.05, 0.67)	1.02	(0.27, 3.77)
*Perceived Social Benefits*								
Looking cool	0.98	(0.16, 6.12)	20.60 *	(1.46, 292.70)	0.91	(0.06, 13.39)	-	-
More friends	3.04 *	(1.01, 9.22)	2.73	(0.53, 14.10)	3.04	(0.62, 14.82)	-	-
Dealing with problems	1.04	(0.09, 11.60)	8.44	(0.78, 91.20)	14.32 *	(1.81, 113.28)	12.90 *	(1.02, 164.80)
*Normative Expectations*								
Perception of friends’ use	2.49 **	(1.49, 4.18)	2.26	(0.94, 5.50)	2.89 **	(1.30, 6.42)	5.13 **	(1.80, 14.60)
Perception of peer use	1.22 *	(1.04, 1.42)	1.11	(0.84, 1.47)	1.22	(0.93, 1.60)	1.19	(0.83, 1.71)

Note: * *p* < 0.05; ** *p* < 0.01; *** *p* < 0.001.

## Data Availability

The data presented in this study are available on request from the corresponding author.
